# Tips and Tricks for Early Diagnosis of Cervico-Vaginal Involvement from Extramammary Paget’s Disease of the Vulva: A Referral Center Experience

**DOI:** 10.3390/diagnostics13030464

**Published:** 2023-01-27

**Authors:** Anna Daniela Iacobone, Maria Elena Guerrieri, Eleonora Petra Preti, Noemi Spolti, Gianluigi Radici, Giulia Peveri, Vincenzo Bagnardi, Giulio Tosti, Angelo Maggioni, Fabio Bottari, Chiara Scacchi, Mariacristina Ghioni

**Affiliations:** 1 Preventive Gynecology Unit, European Institute of Oncology IRCCS, 20141 Milan, Italy; 2 Department of Biomedical Sciences, University of Sassari, 07100 Sassari, Italy; 3 Department of Clinical Sciences and Community Health, University of Milan, 20122 Milan, Italy; 4 Department of Medical Epidemiology and Biostatistics, Karolinska Institutet, 17177 Stockholm, Sweden; 5 Department of Statistics and Quantitative Methods, University of Milan-Bicocca, 20126 Milan, Italy; 6 Dermato-Oncology Unit, European Institute of Oncology IRCCS, 20141 Milan, Italy; 7 Department of Gynecology, European Institute of Oncology IRCCS, 20141 Milan, Italy; 8 Division of Laboratory Medicine, European Institute of Oncology IRCCS, 20141 Milan, Italy; 9 Division of Diagnostic Cytology, European Institute of Oncology IRCCS, 20141 Milan, Italy; 10 Division of Pathology, European Institute of Oncology IRCCS, 20141 Milan, Italy

**Keywords:** vulvar extramammary Paget’s disease (EMPD), cervico-vaginal involvement, atypical glandular cytology, colposcopic-guided biopsy, Paget cells

## Abstract

Cervico-vaginal (CV) localization of extra-mammary Paget’s disease (EMPD) of the vulva is extremely rare. In order to investigate the incidence risk and the pathognomonic clinical and pathological features of this condition, a retrospective analysis was conducted including 94 women treated for vulvar EMPD at the European Institute of Oncology, Milan, Italy, from October 1997 to May 2020. Overall nine patients developed CV involvement from EMPD, with a cumulative incidence of 2.5% (95% CI: 0.5–8.0%) at 5 years, 6.5% (95% CI: 1.9–15.1%) at 10 years and 14.0% (95% CI: 4.8–27.8%) at 15 years, respectively. All cases except one were firstly detected by abnormal glandular cytology. None reported vaginal bleeding or other suspicious symptoms. The colposcopic findings were heterogeneous and could sometimes be misdiagnosed. Cervical and/or vaginal biopsies were always performed for histopathological diagnosis by identification of Paget cells in the epithelium or stroma. Most patients developed invasive EMPD (5/9) of the cervix and/or vagina and underwent hysterectomy with partial or total colpectomy. CV involvement from EMPD should not be underestimated in women with a long-standing history of vulvar Paget’s disease. Liquid-based cytology with immunocytochemistry represents a valuable tool for early diagnosis and should be routinely performed during the required lifelong follow-up.

## 1. Introduction

Extramammary Paget’s disease (EMPD) of the vulva is a rare vulvar neoplasia with an unclear pathophysiology that usually occurs in the apocrine gland-rich skin of post-menopausal Caucasian women [1]. Vulvar EMPD predominantly manifests as an intraepithelial tumor (primary EMPD) but can also appear with stromal invasion or in association with an underlying lower genital tract or distant adenocarcinoma (secondary EMPD) [2].

The clinical presentation is various and includes erythematous, scaly or eczematous plaque on the vulva and perineum with occasional erosions or ulcerations, hypopigmentation and nodules. Itching and burning pain are the most common symptoms. Due to the overlap of signs and symptoms with other vulvar diseases, the diagnosis is confirmed by histological assessment on punch or excision biopsy. It is also well known that EMPD often appears as multifocal and/or with the histological extent of the disease far beyond the visible macroscopic lesion [3]. Moreover, despite surgical excision, local recurrence has been reported in up to 73% of cases and negative resection margins cannot ensure relapse-free survival [4]. On the contrary, Matsuo et al. recently showed that positive surgical margins are significantly associated with an increased risk of local but not distant recurrence [5]. Nevertheless, alternative therapeutic regimes have been advocated over time, including laser excision and ablation, topical therapy with imiquimod, photodynamic therapy and radiotherapy, since multiple surgical instances for recurrences lead to the destruction of vulvar anatomy with psychosocial consequences. 

The cervical and vaginal localization of EMPD has only been described in the case reports as an extremely rare extension of recurrent vulvar EMPD and was firstly described in 1988 by Costello et al. [6]. 

While investigations based on age and anatomical site to distinguish between primary and secondary EMPD are well established, little is known about how to early diagnose cervico-vaginal (CV) localization of EMPD.

The objective of the present study was to investigate the incidence risk of CV involvement from EMPD of the vulva in women referred to a tertiary cancer center and to identify the pathognomonic clinical and pathological features of this rare evolution of vulvar EMPD.

## 2. Materials and Methods

All women affected by EMPD of the vulva and attending the Preventive Gynecologic Unit of the European Institute of Oncology, Milan, Italy, from October 1997 to May 2020, were retrieved from hospital file archives and enrolled in a retrospective analysis.

The local Institutional Review Board approved the study protocol (IEO protocol number UID 2408, date of approval: 22 June 2020) and written informed consent for the use of data for scientific purposes was obtained from all subjects prior to treatment.

Patients were included if the following criteria were met: (a) age at diagnosis of 18 years or older; (b) histologic confirmation of vulvar EMPD; (c) available data regarding follow-up. Patients were excluded in the case of different histology of vulvar neoplasia.

The data regarding clinical and pathological characteristics of the patients were recorded in a dedicated database. 

The histological characteristics of first diagnosis, vulvar recurrence and cervical and/or vaginal localization were retrieved from surgical and pathological reports. All histological diagnoses were conducted by dedicated gynecological pathologists working at the Pathology Division of our Institute. Vulvar EMPD was classified according to the classification of Wilkinson and Brown as either primary, if Paget cells were of cutaneous origin, or secondary, in the case of vulvar skin involvement derived from an internal noncutaneous malignancy. The primary vulvar EMPD was further classified as exclusively intraepithelial (Type 1a), associated with stromal invasion (Type 1b) and as a manifestation of a primary vulvar adenocarcinoma (Type 1c). Secondary vulvar EMPD could be associated with anal or rectal adenocarcinoma (Type 2a), urothelial carcinoma (Type 2b) and distant tumors, including hepatocellular and breast carcinomas (Type 2c) [7]. 

Follow-up was routinely scheduled at the dedicated Vulvar Pathology Clinic of our Institute. 

Apart from primary HPV screening, a pap smear was routinely performed once a year, also in women older than 65 years. In the case of abnormal cytology, women underwent colposcopy with cervical and/or vaginal guided biopsies. When atypical glandular cells were detected, endocervical curettage, endometrial biopsy and transvaginal ultrasound were always performed to rule out the origin of abnormal cells from endocervix, endometrium, ovary or Fallopian tube. If not available, HPV testing was conducted to exclude HPV-related disease. 

A dedicated database was prospectively filled at each follow-up visit.

Therapeutic approaches, including surgery and alternative treatments such as topical therapy with imiquimod, photodynamic therapy and radiotherapy, as well as the type and timing of any persistence or recurrence, invasive disease and cervico-vaginal (CV) localization of EMPD were registered. 

To improve the accuracy of the survival data, telephone interviews and consultation of civil registries were allowed in the case of patients lost to follow-up.

### Statistical Analysis

Patients’ history, characteristics at diagnosis, therapeutic pathway and follow-up occurrences were summarized as the count and percentage for the categorical variables, as a mean and range for the continuous variables, and as the median and range for the skewed variables. The cumulative incidence function (CIF) of the CV localization was computed considering death as a competing event. The overall survival was estimated by the Kaplan–Meier (KM) method, and a survival curve was represented. Statistical analyses were performed using SAS statistical software version 9.4 (SAS Institute Inc., Cary, NC, USA).

## 3. Results

After applying the inclusion and exclusion criteria, 94 women affected by vulvar EMPD and treated at the European Institute of Oncology, from October 1997 to May 2020, were selected for our retrospective analysis.

The mean age of patients at the time of first diagnosis was 63.3 years (range: 31–88) and the median follow-up time was 7 years + 10 months (range: 2 months–30 years + 7 months).

The main clinical and pathological characteristics of the enrolled women at first diagnosis and during follow-up are shown in Table 1.

Most of the patients (81%) were affected by intraepithelial EMPD (Type 1a) at first diagnosis. Invasive EMPD occurred in only 36% of cases, including 17 patients diagnosed at first occurrence and 17 during follow-up. The histology of the invasive EMPD patients is listed in Table 1.

Persistence or recurrence was very common, taking place in 86% of cases and, thus, often requiring multiple surgical instances (median: 2; range: 0–11). The histology of persistence/recurrence of EMPD is detailed in Table 1. Alternative treatments were applied, especially in the case of relapse, including local therapy with imiquimod (63%), photodynamic therapy (5%) and radiotherapy (12%).

**Figure 1:**
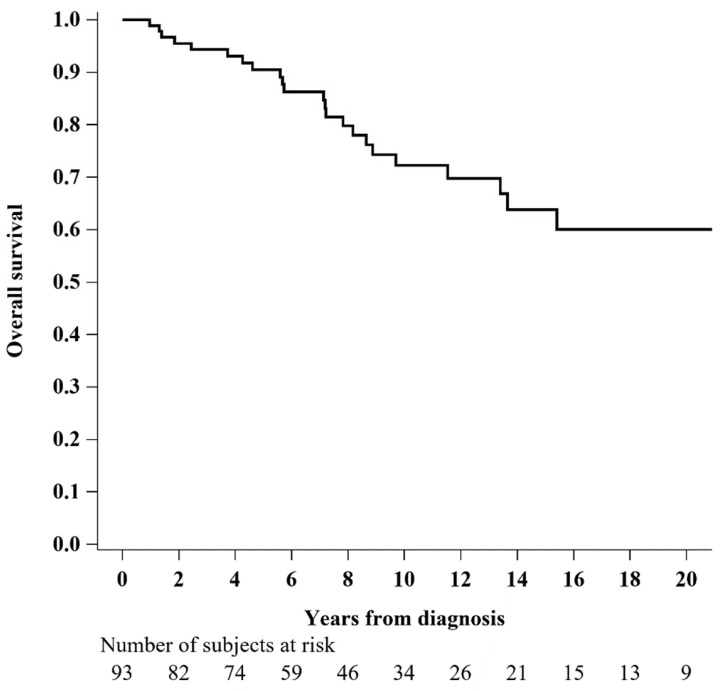
Overall survival of women affected by vulvar EMPD (N = 93).

After excluding one patient with an unknown death date, the 5 year overall survival was 90.5% (95% CI: 81.8–95.1%), as shown in Figure 1.

**Figure 2:**
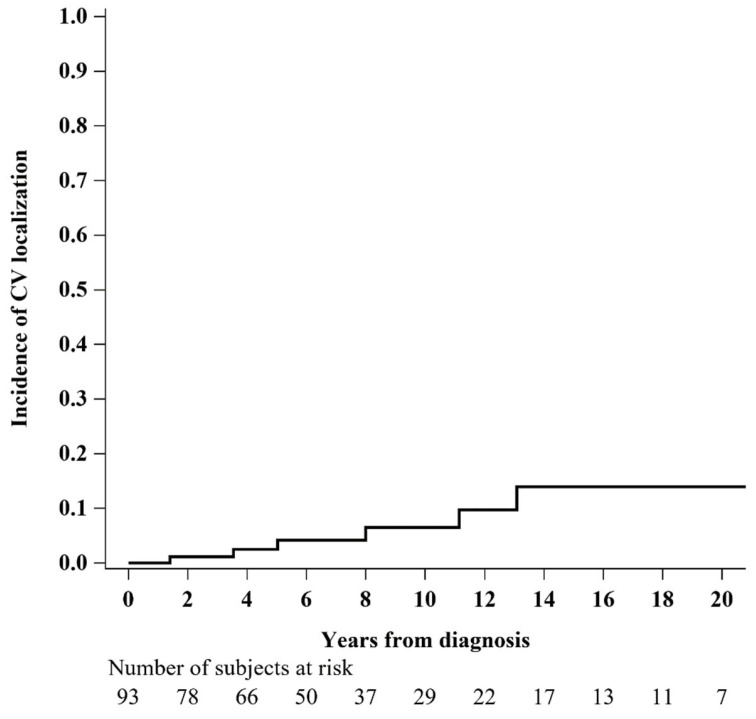
Cumulative incidence function of cervico-vaginal (CV) localization of vulvar EMPD, considering death as a competing event (N = 93).

Overall, nine women developed CV localization of EMPD, with a cumulative incidence of 2.5% (95% CI: 0.5–8.0%) at 5 years, 6.5% (95% CI: 1.9–15.1%) at 10 years and 14.0% (95% CI: 4.8–27.8%) at 15 years, respectively (Figure 2).

The main characteristics of women who developed CV involvement from vulvar EMPD are reported in Table 2, including histology at diagnosis and at vulvar recurrence and the timing and type of CV localization. The majority of women (6/9) showed an intraepithelial vulvar EMPD (Type 1a) at first diagnosis, but three of them developed an invasive disease (Type 1b) at recurrence. The CV localizations occurred after a median time of 133 months (range: 16–334) from the first diagnosis of vulvar EMPD. All cases except one were firstly detected by abnormal pap smear and histologically confirmed by cervical and vaginal colposcopic-guided biopsies. 

Most patients (5/9) developed invasive EMPD of the cervix and/or vagina. In the case of invasive or unusual disease, magnetic resonance imaging (MRI) of the lower abdomen and total body positron emission tomography (PET) were performed to rule out pelvic node or distant metastases. Interestingly, one woman with vulvar EMPD Type 1a at the onset developed invasive cervico-vaginal involvement with node metastasis after 251 months from the initial diagnosis. CV intraepithelial EMPD occurred in only two patients, among which one later developed invasive EMPD of the urethra. Two CV localizations manifested with other associated diseases: invasive urothelial carcinoma and invasive mucinous intestinal-type adenocarcinoma with node metastasis.

Most of the women underwent hysterectomy with partial or total colpectomy based on the site of extravulvar EMPD localization. The patient with node metastases was treated with chemotherapy in association with anti-HER2 (human epidermal growth factor 2)-targeted monoclonal antibodies. Instead, the patient diagnosed with cervical invasive mucinous intestinal-type adenocarcinoma refused any treatment because of comorbidities and died nine months after the diagnosis. Radiotherapy was offered to the woman who developed an unresectable and advanced form of invasive EMPD of the urethra.

### 3.1. Clinical Features 

None of the women reported suspicious symptoms, such as vaginal bleeding or discharge. Almost all cases were detected by abnormal glandular cytology. An HPV DNA test resulted negative in all patients. Endometrial biopsy and transvaginal ultrasound ruled out the origin of abnormal glandular cells from endometrium, ovary and Fallopian tube in all cases.

### 3.2. Cytology

**Figure 3:**
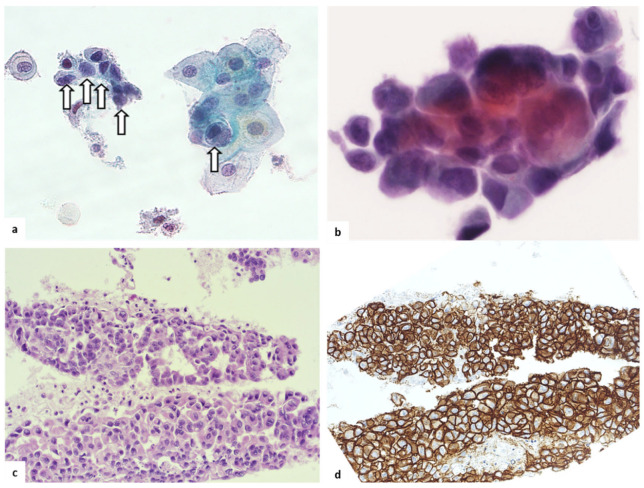
Cervical cytology of EMPD: (**a**,**b**) liquid-based cytology; (**c**,**d**) cell block. Note the (arrow) Paget cells as round or columnar cells with an increased N/C ratio (**a**, 20×), arranged in small clusters (**b**, 40×) on liquid-based cytology and highlighted on the cell block (**c**, 20×) by HER2 expression (**d**, 20×).

Liquid-based cervical cytology revealed the presence of atypical or frankly malignant glandular cells in eight out of nine cases affected by CV localization of vulvar EMPD. The Paget cells were round to columnar with an increased nuclear/cytoplasmic (N/C) ratio and vacuolated cytoplasm. Since HER2 is frequently expressed in genital and anal EMPD (15–60% of cases) [8], a cell block was set-up with residual cellularity and HER2 expression explored by immunocytochemistry in four cases in order to support the diagnosis (Figure 3).

### 3.3. Colposcopy

Colposcopy with endocervical curettage and guided ectocervical and/or vaginal biopsies was performed in all patients diagnosed with atypical glandular cells on pap smear in order to assess the nature of the atypical cells, the extent of the disease and make the best therapeutic decision. 

**Figure 4:**
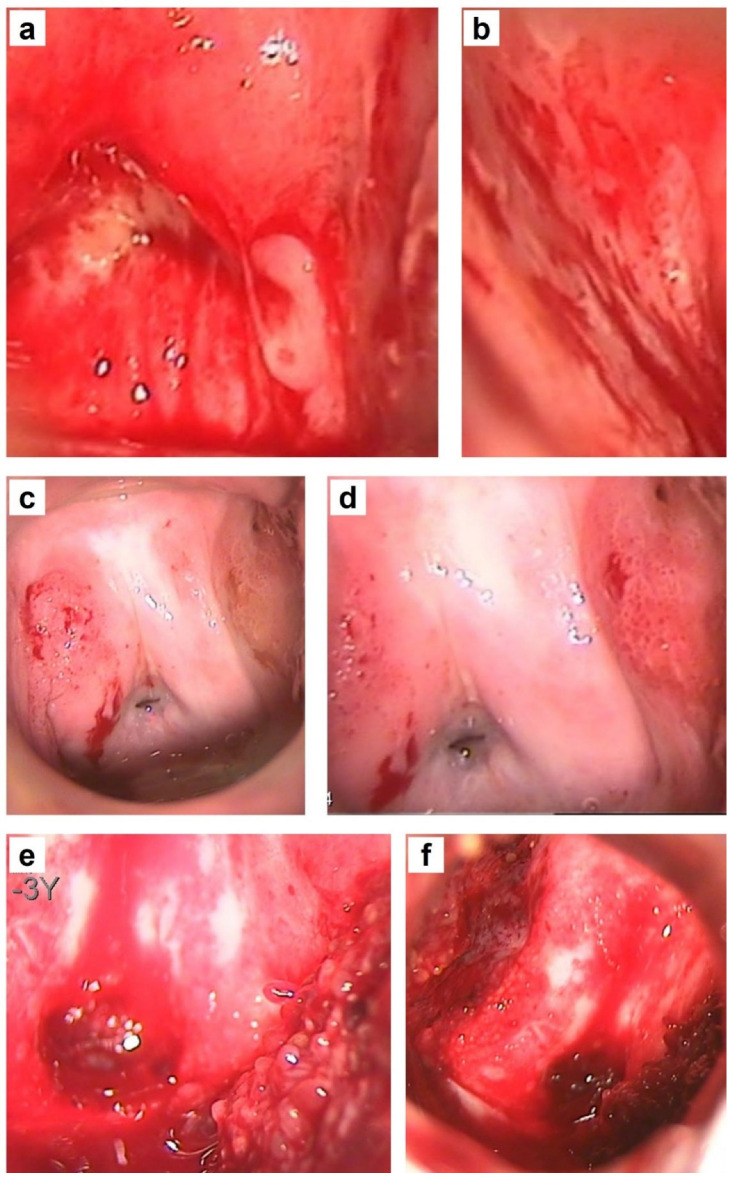
Heterogeneous colposcopic findings in women diagnosed with CV involvement from vulvar EMPD: (**a**,**b**) Dense acetowhite epithelium with sharp borders and coarse punctuation: inside the transformation zone of the cervix and in the left vaginal fornix (**a**); in the middle third of the left vaginal wall (**b**). (**c**,**d**) Micropapillary lesions with ridge sign and coarse punctuation: outside the transformation zone of the cervix (**c**); in the upper third of the left vaginal wall (**d**). (**e**,**f**) Large papillae with an irregular surface and fragile vessels: in the left vaginal wall (**e**); in the right and left vaginal walls with a wide extension (**f**).

The colposcopic findings were heterogeneous in our CV localizations of EMPD and could sometimes be misdiagnosed as high-grade intraepithelial squamous lesions. After acetic acid wash, major abnormal colposcopic findings were revealed in all patients. However, some cases showed dense acetowhite epithelium with a sharp border, whereas other cases appeared as micropapillary lesions with ridge sign or large papillae with irregular surface and fragile vessels. Coarse punctuation was common in almost all cases of CV EMPD. The location of lesions could be inside or outside the transformation zone of the cervix and in the vaginal fornices or walls. Most of the lesions were multifocal and with a wide extension (Figure 4).

### 3.4. Histopathological Diagnosis

**Figure 5:**
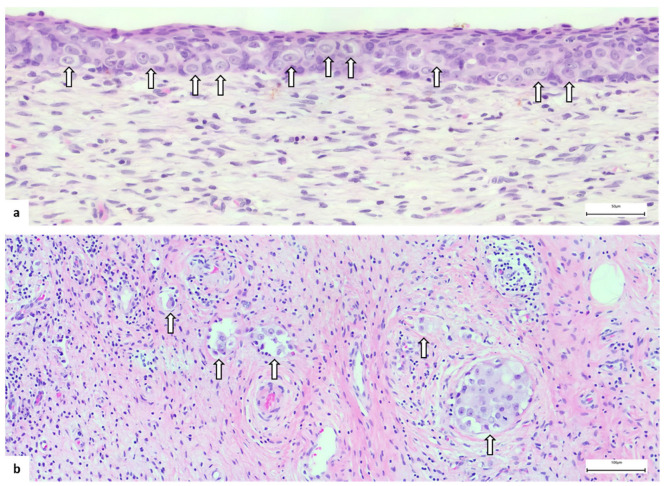
Cervical EMPD: (**a**) intraepithelial and (**b**) invasive. Note the (arrow) Paget cells, with clear cytoplasm and prominent nucleoli, arranged as single cells or little clusters in the ectocervical epithelium (**a**) and as gland-like structures in the cervical stroma (**b**).

As in vulvar EMPD, the diagnostic clue in CV localization is the presence of Paget cells in the epithelium or stroma. Paget cells are large cells with abundant pale cytoplasm, large vesicular nuclei and prominent nucleoli, arranged as single cells or cell clusters throughout the epithelium and/or in the stroma (Figure 5). The diagnosis is not so difficult in cases with a long history of vulvar EMPD, but it could represent a challenge in some instances. Among the differential diagnoses, it is mandatory to consider intraepithelial or invasive squamous cells carcinoma and malignant melanoma in the first instance and to take into account the involvement by an internal regional cancer (colon or urinary bladder, mainly). A diagnostic immunohistochemical panel is recommended for excluding EMPD mimics [3], comprising cytokeratin (CK)7, CK20, p63, SOX10 and carcinoembryonic antigen (CEA). EMPD is typical CK7-positive, CK20-positive or negative, p63-negative, SOX10-negative and CEA-positive unlike squamous cell carcinoma which is p63-positive and malignant melanoma which is SOX10-positive. To rule out the possibility of spread from an internal tumor, CDX-2 (negative in EMPD and positive in colon cancer) and uroplakin-III (negative in EMPD and positive in urothelial carcinoma) are helpful.

## 4. Discussion

CV involvement from EMPD occurred in 9.6% (9/94) of women affected by vulvar EMPD and attending the Preventive Gynecologic Unit of the European Institute of Oncology, Milan, Italy, from October 1997 to May 2020.

This rare condition has already been reported by a few case reports in previous years [9,10,11,12,13]. Only Gu at al. reported a higher prevalence (15.6%) of patients with vulvar EMPD who developed CV localization during the course of their disease. However, a potential bias in their results is the limited number (only 19) of women who were retrospectively analyzed and among whom three were diagnosed with CV EMPD after an abnormal pap smear [14]. This could obviously lead to an overestimation of this rare evolution of vulvar Paget’s disease. 

Nevertheless, cervix and/or vagina could be involved more than usually expected, as a direct contiguous extension from the vulva. Indeed, it is already well known that vulvar EMPD could histologically extend beyond the visible lesion, even if primary and intraepithelial [10,11].

In addition, CV EMPD can be incidentally diagnosed on exfoliative cytology smears, though not differentiating between intraepithelial and invasive disease, as widely reported previously in the literature [15,16]. Therefore, a Papanicolaou smear should be routinely performed even in the cases of benign appearance of the cervix and vagina [10]. 

However, when atypical glandular cells are detected on a pap smear, endocervix, endometrium, ovary and Fallopian tube should be always investigated as a potential source, since related malignancies are more common than Paget’s disease [17].

A clinical history of EMPD in women with abnormal glandular cytology could be helpful for pathological diagnosis and is often crucial for a differential diagnosis. Indeed, immunocytochemistry is not usually necessary but represents an additional valuable tool to distinguish Paget cells from high-grade squamous lesions on liquid-based cytology specimens of suspicious glandular lesions in women with known EMPD of the vulva [18].

Although only described by rare case reports [6,10,14], Papanicolaou smear still plays a fundamental role for the early detection of Paget cells in the cervix and/or vagina. This assumption is very noteworthy in our recent time when HPV testing alone has been advocated as the best cost-effective strategy for cervical cancer screening [19,20]. All of our cases of CV EMPD had a negative HPV DNA test result and would not be identified by HPV primary screening. Moreover, in most cases, CV EMPD occurred in women older than 65 years of age when routine screening was usually discontinued. Hence, according to our experience, there is a strong clinical rationale to routinely perform pap smear in older patients with a history of vulvar Paget’s disease.

Colposcopic findings when Paget cells are detected in the pap smear have been reported as normal or minor abnormal in the past literature [10]. In our retrospective analysis, colposcopic findings were major abnormal in all patients with CV localizations of EMPD, but widely heterogeneous and could sometimes be misdiagnosed as high-grade intraepithelial squamous lesions. Thus, colposcopic-guided biopsies are mandatory for full assessment in order to confirm histopathological diagnosis of EMPD after an abnormal glandular cytology, as already suggested by other authors [9,21]. 

Interestingly, according to our experience, the cumulative incidence of CV localization of vulvar EMPD increases with an increasing survival time: 2.5% at 5 years, 6.5% at 10 years and 14.0% at 15 years. The risk of CV EMPD should always be considered in women with longstanding, extensive and recurrent vulvar disease. Long-term follow-up with routine liquid-based cervical cytology is highly recommended.

To the best of our knowledge, the present study is the largest case series of women diagnosed with CV involvement from EMPD of the vulva. Furthermore, this is the first paper that retrospectively analyzes and describes all pathognomonic clinical and pathological features of this condition in order to identify which steps might be useful for early diagnosis by clinicians.

Early diagnosis of this disease manifestation is a key step in choosing the correct therapeutic management. Surgical approach is always the first choice in the case of CV localization of EMPD in the absence of lymph node metastasis. The role of chemotherapy and radiotherapy for this disease is not well defined unlike vulvar squamous cell carcinoma and should be reserved only for unresectable advanced disease and/or with lymph node metastases [22,23]. However, combined chemotherapy and anti-HER2-targeted therapy represents a promising strategy in patients with advanced or recurrent EMPD of the vulva [24].

The limits of this study include selection bias related to the single-center retrospective analysis and the small sample of events, which did not allow for a logistic regression analysis to investigate risk factors for the development and occurrence of CV EMPD.

## 5. Conclusions

CV involvement from EMPD is a rare condition but should not be underestimated in women with a long-standing history of vulvar Paget’s disease. It can be promptly detected by cytology, which is a valuable and reliable tool for early diagnosis and should be routinely performed during the required lifelong follow-up. 

## Figures and Tables

**Table 1:** Clinical and pathological characteristics of women affected by vulvar EMPD at first diagnosis and during follow-up (N = 94).

Variable	N (%)
Histology at diagnosis	
1a	76 (81)
1b	10 (11)
1c	7 (7)
*N+*	*1*
2a	1 (1)
Previous or concurrent cancer	
No	67 (71)
Breast	14 (15)
Endometrial	2 (2)
Bladder–Urethral	3 (3)
Colorectal	1 (1)
Vulvar squamous	4 (4)
Other	3 (3)
Surgery	
No	2 (2)
Yes	92 (98)
Imiquimod	
No	32 (34)
Yes	59 (63)
Missing	3 (3)
Photodynamic therapy	
No	87 (93)
Yes	5 (5)
Missing	2 (2)
Radiotherapy	
No	81 (86)
Yes	11 (12)
Missing	2 (2)
Persistence or recurrence	
No	12 (13)
Yes	81 (86)
Unknown	1 (1)
Histology at persistence or recurrence	
1a	47 (58)
1b	9 (11)
1c	3 (4)
*N+*	*1*
2a	3 (4)
Invasive mammary PD	1 (1)
*N+*	*1*
Unknown histology	18 (22)
Invasive EMPD	
No	60 (64)
Yes	34 (36)
Time of diagnosis of invasive EMPD	
At diagnosis of EMPD	17 (50)
During the follow-up	17 (50)
Histology of invasive EMPD	
1b	19 (56)
1c	11 (32)
*N+*	*1*
2a invasive	2 (6)
*N+*	*2*
Invasive mammary PD	1 (3)
*N+*	*1*
Invasive PD of the urethra	1 (3)
Abnormal pap smear	
No	82 (87)
Yes	11 (12)
Unknown	1 (1)
Cervico-vaginal localization	
No	84 (89)
Yes	9 (10)
Unknown	1 (1)

**Table 2:** Principal characteristics of women with cervico-vaginal (CV) localization of vulvar EMPD (N = 9).

Subject ID	Age at Diagnosis (Years)	Histology of Vulvar EMPD at First Diagnosis	Histology of Vulvar EMPD at Recurrence	Time to Abnormal Cytology (Months)	Time to Diagnosis of CV Localization (Months)	Site of CV Localization	Histology of CV Localization
1	52	1a	1a	155	157	Cervical and vaginal	Intraepithelial EMPD *
2	42	1a	1a	251	260	Cervical and vaginal N+	Invasive EMPD
3	67	1a	1b	94	95	Vaginal	Invasive EMPD
4	59	1a	1a	132	133	Cervical and vaginal	Invasive urothelial carcinoma
5	62	1a	1b	251	255	Cervical and vaginal	Intraepithelial EMPD
6	86	1a	1b	NA	60	Vaginal	Invasive EMPD
7	46	1b	1b	333	334	Cervical and vaginal	Invasive EMPD
8	70	1c	1c	15	16	Cervical and vaginal	Invasive EMPD
9	74	2a	Invasive mucinous intestinal-type adenocarcinoma	30	42	Cervical N+	Invasive mucinous intestinal-type adenocarcinoma

* Developed invasive EMPD of the urethra after CV localization.

## Data Availability

The data presented in this study are available upon request from the corresponding author. The data are not publicly available due to the patients’ privacy restrictions. The data are safely stored in a private database of the European Institute of Oncology, Milan, Italy.
